# Mental Health Crisis and Policing Demand

**DOI:** 10.23889/ijpds.v9i5.2504

**Published:** 2024-09-18

**Authors:** Allison Kurpiel, Susan McVie

**Affiliations:** 1 University of Edinburgh

## Objectives

Police are frequently called to assist in situations where mental ill health, rather than crime, is the primary concern. There is an urgent need to reduce this demand on police by improving appropriate health service delivery. This research examines police interactions in which an individual is identified as being vulnerable due to a mental health concern. We aim to determine the demographic, health, and social factors that are associated with being identified by police as vulnerable due to a mental health concern and assess patterns of health system service use preceding police contact to identify potential points for intervention.

## Methods

A dataset of police records for individuals identified as vulnerable due to a mental health concern is linked to health and social administrative data including: information on mental ill health, alcohol and drug related problems, disabilities and long-term illnesses, Accident and Emergency calls, hospital visits, and demographic characteristics. We describe a cohort of individuals identified by police as vulnerable due to a mental health concern and assess their traits and patterns of healthcare service use compared to the general population.

## Results

The results allow us to better understand who comes into contact with the police while experiencing mental health difficulties and point to the risk factors indicating where to concentrate resources for improved health service delivery.

## Conclusions/Implications

This research will improve partnership working between the police and healthcare services, reduce unnecessary police demand, and improve outcomes for people suffering from mental health crisis.

